# Application of Machine Learning and Emerging Health Technologies in the Uptake of HIV Testing: Bibliometric Analysis of Studies Published From 2000 to 2024

**DOI:** 10.2196/64829

**Published:** 2025-05-22

**Authors:** Musa Jaiteh, Edith Phalane, Yegnanew A Shiferaw, Lateef Babatunde Amusa, Hossana Twinomurinzi, Refilwe Nancy Phaswana-Mafuya

**Affiliations:** 1 South African Medical Research Council/University of Johannesburg Pan African Centre for Epidemics Research Extramural Unit, Faculty of Health Sciences, University of Johannesburg, Auckland Park Bunting Road Campus, PO Box 524, Auckland Park, Johannesburg, 2006, South Africa, 27 632376425, 27 115591496; 2 Department of Statistics, Faculty of Science, University of Johannesburg, Johannesburg, South Africa; 3 Center of Applied Data Science, University of Johannesburg, Johannesburg, South Africa; 4 Department of Statistics, University of Ilorin, Ilorin, Nigeria

**Keywords:** bibliometric analysis, machine learning, mHealth, technology, HIV prevention, HIV testing, application, bibliometric, HIV, epidemiology, AIDS, self-testing, sexually transmitted disease, quantitative, bibliography, content analysis, mobile health

## Abstract

**Background:**

The global targets for HIV testing for achieving the Joint United Nations Programme on HIV/AIDS (UNAIDS) 95-95-95 targets are still short. Identifying gaps and opportunities for HIV testing uptake is crucial in fast-tracking the second (initiate people living with HIV on antiretroviral therapy) and third (viral suppression) UNAIDS goals. Machine learning and health technologies can precisely predict high-risk individuals and facilitate more effective and efficient HIV testing methods. Despite this advancement, there exists a research gap regarding the extent to which such technologies are integrated into HIV testing strategies worldwide.

**Objective:**

The study aimed to examine the characteristics, citation patterns, and contents of published studies applying machine learning and emerging health technologies in HIV testing from 2000 to 2024.

**Methods:**

This bibliometric analysis identified relevant studies using machine learning and emerging health technologies in HIV testing from the Web of Science database using synonymous keywords. The Bibliometrix R package was used to analyze the characteristics, citation patterns, and contents of 266 articles. The VOSviewer software was used to conduct network visualization. The analysis focused on the yearly growth rate, citation analysis, keywords, institutions, countries, authorship, and collaboration patterns. Key themes and topics were driven by the authors’ most frequent keywords, which aided the content analysis.

**Results:**

The analysis revealed a scientific annual growth rate of 15.68%, with an international coauthorship of 8.22% and an average citation count of 17.47 per document. The most relevant sources were from high-impact journals such as the *Journal of Internet Medicine Research*, *JMIR mHealth and uHealth*, *JMIR Research Protocols*, *mHealth*, *AIDS Care-Psychological and Socio-Medical Aspects of AI*, and *BMC Public Health*, and *PLOS One*. The United States of America, China, South Africa, the United Kingdom, and Australia produced the highest number of contributions. Collaboration analysis showed significant networks among universities in high-income countries, including the University of North Carolina, Emory University, the University of Michigan, San Diego State University, the University of Pennsylvania, and the London School of Hygiene and Tropical Medicine. The discrepancy highlights missed opportunities in strategic partnerships between high-income and low-income countries. The results further demonstrate that machine learning and health technologies enhance the effective and efficient implementation of innovative HIV testing methods, including HIV self-testing among priority populations.

**Conclusions:**

This study identifies trends and hotspots of machine learning and health technology research in relation to HIV testing across various countries, institutions, journals, and authors. The trends are higher in high-income countries with a greater focus on technology applications for HIV self-testing among young people and priority populations. These insights will inform future researchers about the dynamics of research outputs and help them make scholarly decisions to address research gaps in this field.

## Introduction

HIV remains a global public health burden, affecting more than 39 million people living with HIV, with approximately 60% of people living with HIV living in sub-Saharan Africa (SSA) [1,2]. HIV/AIDS has claimed 42.3 million lives since the beginning of the epidemic and 630,000 lives in 2023 alone [1]. In addition, the continuous transmission and heightened rates of new HIV infections across different countries remain a significant concern [1,2]. There have been ongoing efforts to curb HIV as a public health threat, among which is the Joint United Nations Programme on HIV/AIDS (UNAIDS) goal of ending AIDS by 2030 [3]. UNAIDS and the World Health Organization (WHO) highlight the significance of prioritizing HIV testing for the realization of this goal [3,4]. Getting tested is the only way to identify people living with HIV for early initiation of antiretroviral therapy to reduce HIV transmission rates [3,4].

Current trends indicate a significant gap in the uptake of HIV testing globally, as only 86% of people living with HIV knew their HIV status by the end of 2023 [2]. This leaves approximately 5.4 million people living with HIV untested, which falls short of the UNAIDS interim target of testing 95% of people living with HIV by 2025 [2,3]. Conventional HIV testing and prevention programs have shown progress, but they fail to address stigma and social discrimination among key populations (KPs) [2-5]. Due to this challenge, HIV testing rates remain low among KPs, who bear the highest burden of HIV incidence [2,5]. In response to this issue, the WHO released updated comprehensive guidelines in 2016 to improve HIV testing coverage while using new strategies, including HIV self-testing (HIVST) [5]. The guidelines are continuously updated to integrate emerging technologies, providing more convenient options for HIV testing and helping to fast-track the UNAIDS 2030 goal.

The emergence of technology-driven health care interventions in recent years has resulted in remarkable advancements, especially in the realm of HIV testing [6]. Among these innovations, machine learning emerged as a noteworthy tool for enhancing the efficacy, accuracy, and accessibility of HIV testing services [7-10]. Machine learning involves the use and development of computer systems that can learn and adapt without explicit instructions by using algorithms to analyze and draw conclusions from data patterns [10].

Machine learning can improve HIV testing in a variety of ways. This methodology accurately predicts latent factors influencing HIV testing by analyzing complex data that could not be explored by the common approaches. Precisely, machine learning identifies individuals at the highest risk of HIV for tailored and cost-effective HIV testing programs to optimize resource allocation [7-11]. This is extremely beneficial, especially for low-income countries with limited resources to combat high HIV rates and other prevailing health issues. A study by Xu et al [11] confirmed that machine learning models such as logistic regression, support vector machines, random forest (RF), Extreme Gradient Boosting (XGBoost), elastic net, and k-nearest neighbor, among other algorithms, were reliable in predicting future HIV risk using complex datasets. Similarly, a retrospective analysis conducted in the United States [12] discovered that RF models seem promising for identifying important predictors of HIV testing uptake. The model’s integration into future HIV prevention and treatment research, as well as intervention program evaluations, was highly encouraged [12]. Datasets from 4 SSA countries were analyzed by XGBoost, support vector machines, RF, elastic net, k-nearest neighbor, and light gradient boosting machine algorithms to accurately identify high-priority groups susceptible to HIV for enhancing targeted HIV testing [7].

Recently, more advanced machine learning technologies have emerged as a game changer for HIV testing concerns such as privacy and accessibility issues with high rates of false positivity/negativity. For instance, the integration of HIVST into machine learning, digital health, and mobile devices is gaining popularity, especially among KPs who usually avoid public services due to stigma [13]. Consistently, the WHO recommends HIVST as a convenient and discrete choice for HIV testing, citing its dependability and excellent performance as key factors for its success [5]. In 2017, van Heerden et al [14] installed artificial intelligence (AI) conversational agents into Android devices to facilitate HIVST and counseling. It was interesting to find out that the South Africans who participated in the study felt the device was more convenient for HIV counseling and testing than the usual procedure. Mobile health (mHealth), which involves using mobile devices to enhance health care services and research, has proven advantageous when incorporated with machine learning [15]. A machine learning mHealth technique was used to detect false-positive HIV test results in another South African study [15]. The technology demonstrated high levels of sensitivity (97.8%) and specificity (100%) compared to conventional visual interpretations of HIV rapid diagnostic tests [15]. Similar advancements were made by [16,17] with their HealthPulse AI technologies, which outperformed human interpretation of HIV test results and provided an opportunity to reduce mental health disturbances associated with false-positive test results.

Leveraging machine learning methods and health technologies can improve HIV testing outcomes by prioritizing high-risk individuals and enhancing the accuracy of HIV testing devices. Despite these advancements, a research gap remains regarding the integration of machine learning techniques into HIV testing interventions. This bibliometric analysis aims to address this gap by examining trends in published studies, journal performances, and collaboration networks among researchers, institutions, and countries regarding using machine learning and emerging health technologies in HIV testing from 2000 to 2024. By identifying key patterns and gaps, the findings will inform future research directions and support more effective implementation of machine learning and emerging health technologies in HIV testing.

## Methods

### Study Approach and Search Strategy

This bibliometric analysis was conducted to understand trends, patterns, and collaboration networks in published studies focused on using machine learning and emerging health technologies in HIV testing from 2000 to 2024. A search string was developed using synonyms of “machine learning” OR “health technologies” and “HIV testing” with Medical Subject Headings (MeSH) terms in PubMed and published keywords from related studies. The articles used for this bibliometric analysis were gathered from the Web of Science (WoS) database. The WoS is one of the world’s top databases for scientific research and citation data, encompassing a wide range of areas such as medicine and health care [18-20]. It has been recognized as a legitimate platform for bibliometric analysis [19-23].

The search query was entered into the WoS search box and filtered by topics, which captured a total of 708 articles at the initial search, and the article list was refined by the inclusion and exclusion criteria listed in Table 1. The study analyzed 266 articles that met the inclusion criteria.

**Table 1.** Inclusion and exclusion criteria.

	Inclusion criteria	Exclusion criteria
Document type	Original and review articles	Proceedings, early access, letters, meeting abstracts, letters, editorial materials, data papers, retracted materials, and corrections.
Years of publication	January 2000 to December 2024	Before January 2000
Language	English	Catalan, French, German, and Spanish
Context	Machine learning and emerging health technologies in HIV testing	Machine learning and emerging health technologies in pre-exposure prophylaxis, antiretroviral therapy, postexposure prophylaxis, voluntary medical male circumcision, HIV education, and unrelated topics.
Research area	Human medicines and health care–related subjects	Religion, government law, history, philosophy of science, arts, humanities, and other topics

The search string that was used to retrieve studies that used machine learning and emerging health technologies in HIV testing from the WoS is detailed here:

(“HIV testing” OR “AIDS Testing” OR “HIV Diagnosis” OR “Rapid HIV testing” OR “HIV Self-Testing” OR “HIV Screening” OR “Human Immunodeficiency Virus Testing” OR “HIV counsel$ing and testing”)

AND

(“Machine Learning” OR “Artificial Intelligence” OR “Unsupervised Learning” OR “Supervised Learning” OR “Model* Algorithm*” OR “Deep Learning” OR “Neural Network*” OR “Support Vector Machine*” OR “Random Forest” OR “Decision Tree*” OR “Convolutional Neural Networks” OR “Recurrent Neural Networks” OR “artificial neural networks” OR “LASSO” OR “XGBoost” OR “ensemble learning” OR “reinforce* learning” OR “Naive Bayes*” OR “AI chatbot” OR “Large language model*” OR “principal component analysis” OR “natural language processing” OR “Health technology” OR “health informatics” OR “Bayesian network*” OR “mHealth” OR “Digital Health” OR “eHealth” OR “Video gam*” OR “Telehealth” OR “Telemedicine” OR “mobile app*”)

### Inclusion and Exclusion Criteria

The inclusion and exclusion criteria outlined in Table 1 were carefully selected to ensure a relevant and comprehensive dataset. Studies were considered for this bibliometric analysis if they applied machine learning and health technologies in HIV testing, diagnosis, or predictive models. Articles that used machine learning and emerging health technologies in other HIV interventions, such as treatment and education programs, were excluded. Moreover, the analysis focused on original and published studies between January 2000 and December 2024. The criteria used in this study were to align the focus of this analysis with the study objective.

### Data Processing and Analysis

#### Data Cleaning and Screening

The bibliographic data were initially cleaned to remove duplicates and exclude studies that fell outside the scope of this study (refer to Table 1). Filters on the WoS database were used to remove articles published before 2000, studies conducted in languages other than English, and studies that were not original or reviewed. Of the 356 articles, 342 remained in the sample and were exported into the Covidence web software for screening. The Covidence software automatically removed 5 duplicates, and 2 independent reviewers (MJ and EP) conducted a comprehensive screening to retain eligible articles in the sample. Articles were screened by title and abstract, and 56 ineligible articles were removed. The full text of the remaining articles was also screened; of these, 266 were selected for the final sample for analysis. The two reviewers independently screened the articles at each stage and resolved disagreements through consensus. The screening process is presented in the Results section.

#### Data Analysis

This bibliometric analysis was performed using the Biliometrix R package (version 4.3.2; Posit Software, PBC) [24], while the VOSviewer software (version 1.6.20) was used for network visualization.

After the data were screened, cleaned, and refined according to the inclusion criteria, 266 references were exported from Covidence as CSV files and exported to Zotero (open-source bibliographic software developed by the global community) for conversion into retained and converted plain text (TXT) files, acceptable file format for the bibliometric software. Thereafter, the downloaded TXT file was uploaded into the Bibliometrix online software (Biblioshiny) [24], an R package (version 4.3.2; Posit Software, PBC). The cleaned bibliographic data was loaded into Biblioshiny to conduct performance analysis and science mapping, and VOSviewer aided in the network visualization.

### Performance Analysis

Performance analysis examines the output and impact of research constituents by quantifying their citation and publication-related metrics [19,20]. It summarizes the characteristics of studies by assessing their productivity (number of publications), impact (citations), and contributors (authors, journals, countries, institutions, subject areas, etc) [19,20,25]. This study first provides a descriptive summary of the selected articles and then presents metrics such as the annual number of scientific productions, top-cited articles, and most productive authors, journals, affiliations, and countries in the field of machine learning in HIV testing.

Various metrics were used to measure the bibliographic sample’s productivity, influence, and impact. For instance, h-index and m-index were used to evaluate the performance of influential authors and journals. According to previous studies [23,25,26], the h-index is referred to as the “highest number of h-index, such that the individual has published ‘h’ papers that each has been cited ‘h’ times.” This means that if an author has published 20 articles and each is cited 12 times, the author’s h-index is 12 [26]. The m-index is a variation of the h-index, showing the h-index per year since the author’s first publication [23]. These two metrics are suitable for measuring the impact of authors and journals [23,26]; thus, they were calculated in this study using the Bibliometrix software. Additionally, the number of global citations for the articles was also used to examine their impact. We further assessed the performances of the authors’ countries and affiliations using the frequency and number of publications on studies related to machine learning in HIV testing between 2000 and 2024.

### Science Mapping

Science mapping, on the other hand, investigates the relationships between research constituents by analyzing the topic’s conceptual, intellectual, and social structure [19,20]. We performed various analyses, including co-citation analysis of cited references, co-occurrence analysis of author keywords, and coauthorship analysis (international collaboration networks). These analyses explore the collaboration patterns between publications, citations, authors, institutions, and countries, as well as describe existing and future relations among topics [20]. These networks explain the research area’s conceptual, intellectual, and social structures, forming the basis for developing key themes for this study. The VOSviewer software (version 1.6.20) provides a visualized and comprehensive understanding of the relationships between different networks within the dataset [20]. Normalization was applied at full counting for co-citation, co-occurrence, and international collaboration analyses. A maximum of 25 countries per document and a minimum of 5 documents per country were established as eligibility criteria, resulting in 53 eligible countries, of which 13 met the collaboration threshold. A total of 598 institutions appeared no more than 25 times in each document, with a minimum requirement of 10 documents per institution; ultimately, 16 institutions met this threshold. The minimum citation requirement for each cited reference was set at 15, and 11 of 8911 cited references satisfied this criterion. Additionally, with a minimum of 10 occurrences required per keyword, 19 of 700 keywords achieved the threshold for co-occurrence analysis. The research constituents, such as country, institution, keyword, author, and document source, were each represented by network nodes. The size of the nodes represents the occurrence frequencies, while the thickness of the links between the nodes determines the strength of the association between networks [23]. These analyses generated key concepts and thematic areas explored in the content analysis. Clustering was used to identify themes from author keywords and the names of the authors’ institutions, as well as country names, within the same cluster in each network. The cluster size was set to 1 with a resolution of 1.00.

### Content Analysis

The study explores the content of articles with the authors’ most frequent keywords, which are also within the scope of the key themes generated by the keywords. The word cloud and co-occurrence analyses aided this process. By analyzing the authors’ co-occurring keywords, we examined various clusters of relevant keywords that formed the basis of the content analysis. The content analysis aided in gaining deeper insights into the patterns and dynamics of the intersection between machine learning/health technologies and HIV testing, as well as suggested future directions from the identified gaps. A qualitative summary of the content analysis presented various topics, which can be found in the Results section.

### Ethical Considerations

This study forms a part of a doctoral study by the first author (MJ) titled “Integration of machine learning algorithms to predict HIV testing associations using repeated cross-sectional survey data in an adult South African population: An HIV testing predictive model.” The study was reviewed and approved by the University of Johannesburg (UJ) Research and Ethics Committee (REC; ethics approval number REC-2725‐2024). Since no primary data were collected, the UJ REC gave a waiver for informed consent. Furthermore, the doctoral study falls under an umbrella project funded by the South African Medical Research Council (SAMRC) under the SAMRC/UJ Pan African Centre for Epidemics Research (PACER) Extramural Unit, titled “Harnessing big heterogeneous data to evaluate the potential impact on HIV responses among the key populations in generalized epidemic settings in Sub-Saharan Africa” (ethics approval number REC-1504‐2022).

## Results

**Figure 1.**
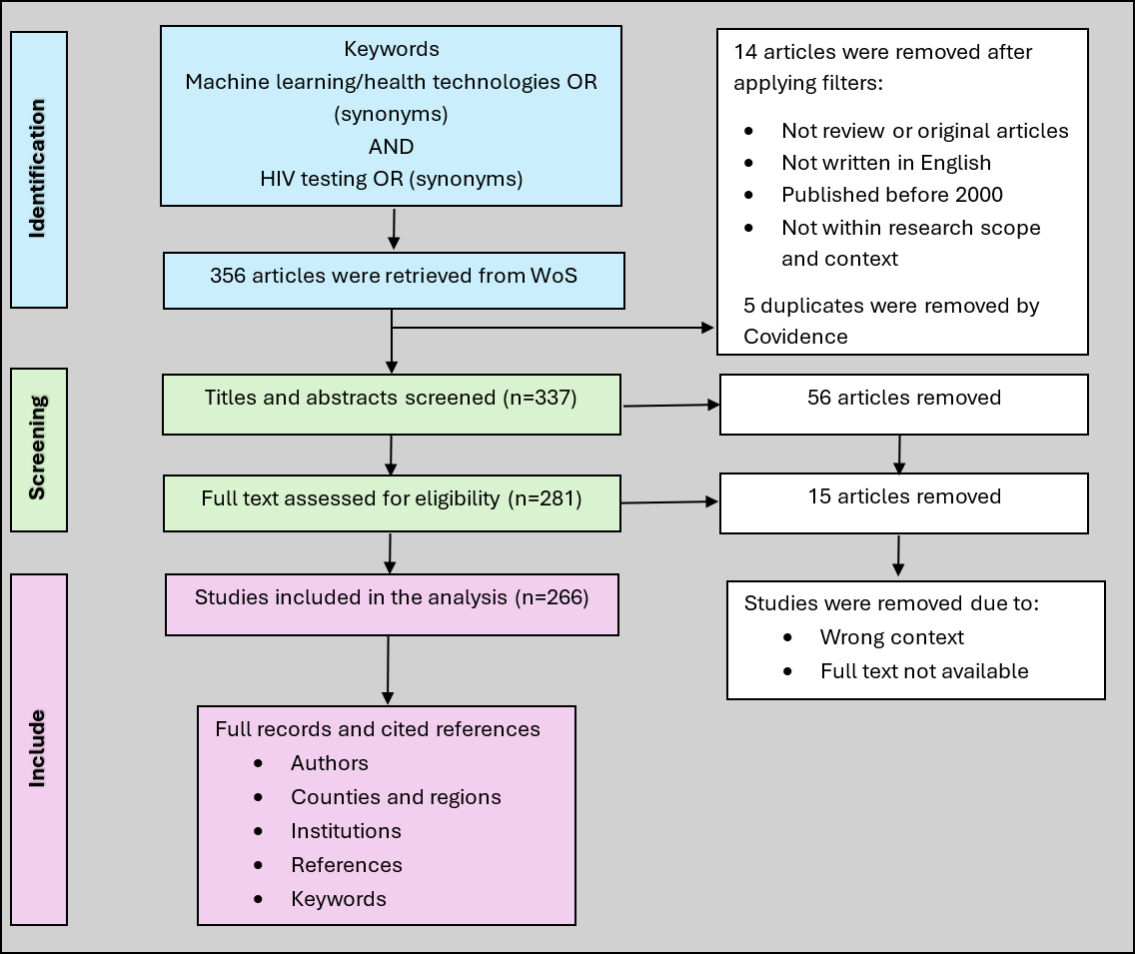
Flowchart showing the search strategy and bibliometric analysis process. WoS: Web of Science.

This study analyzed 266 articles following a comprehensive screening as shown in Figure 1.

### Performance Analysis: Characteristics of the Study Sample

Table 2 summarizes the main characteristics of the articles included in this bibliometric analysis. The analyzed bibliographic dataset comprised 266 articles from 96 sources with 1658 authors and 8918 references. Most (91%) of the documents were original articles, while 9% were review articles. The results show a steady increase in the scientific production of studies using machine learning and emerging health technologies in HIV testing between 2000 and 2024, with an annual growth rate of 15.68%.

**Table 2.** Main information of the extracted bibliographic data of published articles on machine learning in HIV testing from 2000 to 2024.

Description	Results
**Main information about data**	
Time span (years)	2000-2024
Sources (journals, books, etc), n	96
Documents, n	266
Annual growth rate, %	15.68
Document average age (years)	3.85
Average citations per document, n	17.47
References, n	8918
**Document contents**	
Keywords Plus (generated by the indexing database), n	563
Authors’ keywords, n	700
**Authors**	
Authors, n	1658
Authors of single-authored documents, n	2
**Authors’ collaboration**	
Single-authored documents, n	2
Coauthors per document, n	8.22
International coauthorships, %	42.48
**Document types**	
Original articles, n	241
Reviews, n	25

**Figure 2.**
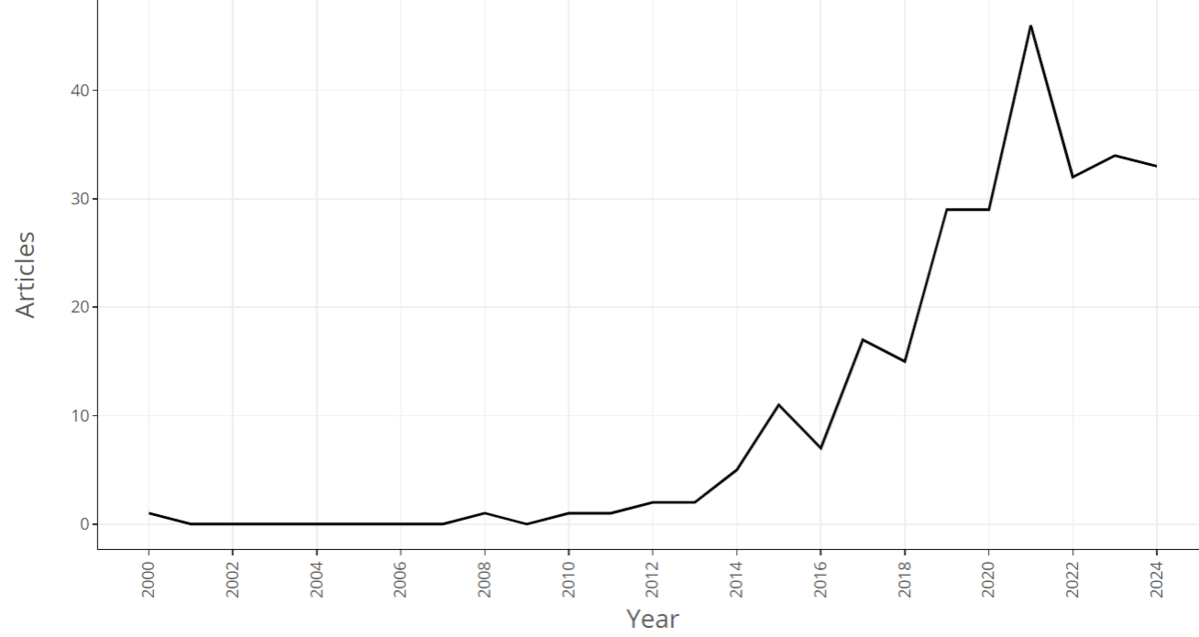
Annual scientific production of articles focused on the application of machine learning in HIV testing.

Figure 2 highlights that annual scientific production in this field became more rapid between 2013 and 2021, but research outputs were comparatively low before 2013. The highest number of publications was recorded in 2021.

The output in Table 3 presents the top 10 most productive and influential journals that publish studies focused on machine learning and emerging health technologies in HIV testing–related topics. Among them, *AIDS and Behavior* ranked at the top of the list, marked by the highest h-index (10), number of citations (494), and number of publications (19). Other sources that are very productive and influential in this research area are *Journal of Medical Internet Research* (h-index=10), *JMIR mHealth and uHealth* (h-index=9), *JMIR Research Protocols* (h-index=8), *mHealth* (h-index 8), *AIDS Care-Psychological and Socio-Medical Aspects of AI* (h-index=6), and *BMC Public Health* (h-index=6), *PLOS One* (h-index=6), *AIDS Education and Prevention* (h-index=5), and *JMIR Formative Research* (h-index=5).

**Table 3.** Top 10 most productive publication sources by h-index.

Source	H-index	M-index	Total citations	Number of publications	Year of first included study
*AIDS and Behavior*	10	0.909	494	19	2014
*Journal of Medical Internet Research*	10	0.909	372	14	2014
*JMIR mHealth and uHealth*	9	0.818	310	14	2014
*JMIR Research Protocols*	8	1.333	264	18	2019
*mHealth*	8	1.6	138	10	2020
*AIDS Care-Psychological and Socio-Medical Aspects of AI*	6	0.6	120	7	2015
*BMC Public Health*	6	0.667	120	9	2016
*PLOS One*	6	0.4	160	9	2010
*AIDS Education and Prevention*	5	0.5	144	6	2015
*JMIR Formative Research*	5	1.25	51	12	2021

In Table 4, the top 10 most productive and influential authors were ranked by h-index, and their scores were based on studies related to machine learning and emerging health technologies in HIV testing. Stephenson has the highest h-index (10), followed by Bauermeister and Sullivan, who each scored 9. Additionally*,* Hightow-Weidman (h-index=8), Horvath (h-index=8), Bukusi (h-index=7), and Muessig (h-index=7) are also very influential in this field. Stephenson has a total of 421 citations with 16 publications of articles centered on machine learning (technology) in conjunction with HIV testing.

**Table 4.** Top 10 most productive and influential authors by h-index.

Author	H-index	M-index	Total citations	Number of publications	First publication year of an included study
Stephenson R	11	1	421	16	2014
Bauermeister JA	9	1.125	176	11	2017
Sullivan PS	9	0.818	406	16	2014
Hightow-Weidman L	8	0.1333	226	10	2019
Horvath KJ	8	0.889	163	13	2016
Bukusi EA	7	0.636	388	8	2014
Muessig KE	7	0.7	223	7	2015
Mustanski B	6	0.6	225	7	2015
Tucker JD	6	0.6	403	11	2015
Cohen CR	5	0.455	353	5	2014

In terms of the most impactful and influential articles, Table 5 presents the top 10 papers that have applied machine learning and emerging health technologies in HIV testing–related studies across the globe. A study by Schnall et al [27] published in the *Journal of Medical Internet Research* was the most influential paper with 129 total citations, 11.73 citations per year, and 1.36 normalized total citations.

**Table 5.** Most globally cited studies on machine learning in HIV testing.

Paper details (first author, year, journal)	Digital object identifier (DOI)	TC^a^	TC per year	Normalized TC
Schnall R, 2014, *J Med Internet Res J*	10.2196/jmir.3393	129	11.73	1.36
Zou HC, 2017, *Arch Sex Behav*	10.1007/s10508-016-0709-3	122	15.25	3.40
Odeny TA, 2014, *AIDS*	10.1097/QAD.0000000000000409	116	10.55	1.22
Chamie G, 2016, *Lancet HIV*	10.1016/S2352-3018(15)00251-9	115	12.78	2.37
Rendina HJ, 2014, *AIDS Behav*	10.1007/s10461-013-0573-2	114	10.36	1.20
Jaiswal J, 2019, *Behav Med*	10.1080/08964289.2019.1630357	106	17.67	4.18
Tang WM, 2018, *PLOS Med*	10.1371/journal.pmed.1002645	101	14.43	3.42
Bien CH, 2015, *AIDS Behav*	10.1007/s10461-014-0994-6	97	9.70	2.58
Sullivan PS, 2017, *JMIR mHealth uHealth*	10.2196/mhealth.7199	96	12.00	2.68
Marcus JL, 2019, *Lancet HIV*	10.1016/S2352-3018(19)30137-7	87	14.50	3.43

a TC: total citations.

**Figure 3.**
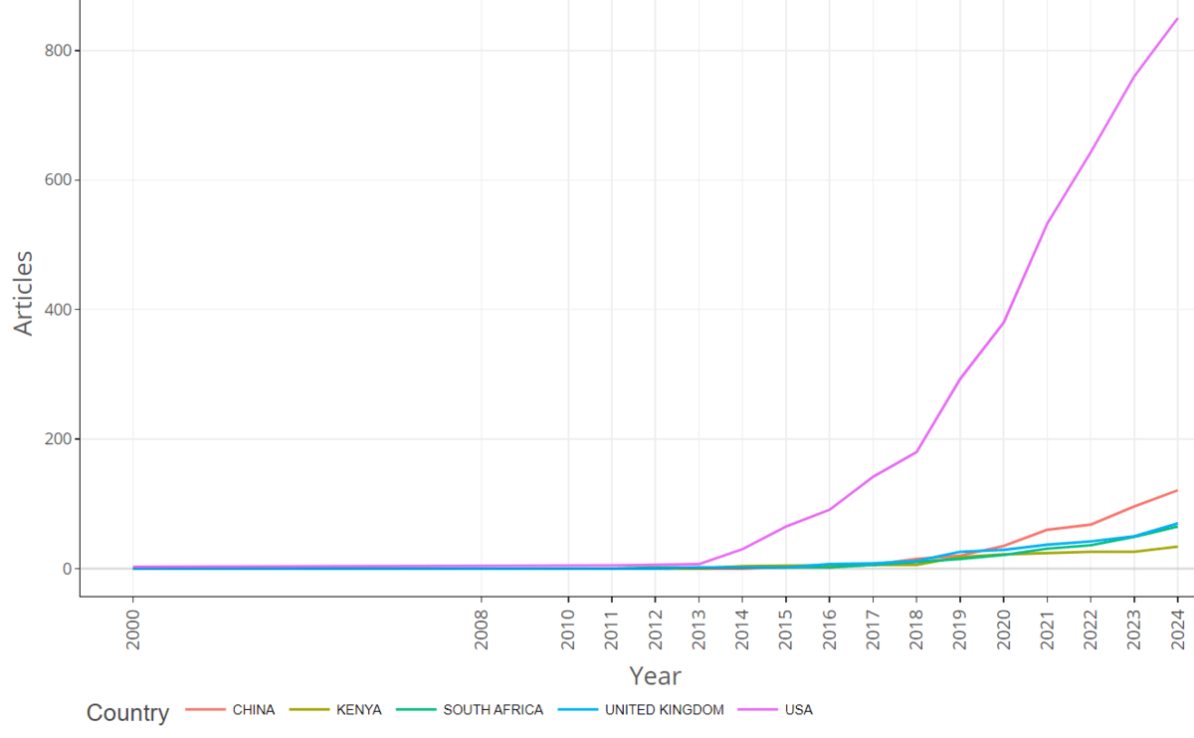
Top 5 countries by annual scientific production. USA: United States of America.

This bibliometric analysis further examines the relevance of institutions and countries worldwide in terms of scientific productivity using publication metrics. Most of the articles included in this study were published by the University of California system (n=67), followed by the University of North Carolina (n=57), the University of North Carolina Chapel Hill (n=52), and the University of Washington (n=48; Table 6). Johns Hopkins University published the fewest studies among the top 10 institutions. As for countries’ productivity, authors’ correspondence information was used, with a total of 30 countries in the sample (Multimedia Appendix 1 [“Most Relevant Countries”] and Table 7). Of the 266 articles within the sample, the United States produced 59% (n=157) of the studies, ranking first (Table 7). There was a notable synchronicity between the United States and other countries regarding the frequency of publications of studies using machine learning in HIV testing from 2000 to 2024. After the United States, the next most publications were from China (n=27, 10.2%), South Africa (n=16, 6%), the United Kingdom (n=10, 3.8%), and Australia (n=7, 2.6%). The top 5 countries’ scientific production in this field was relatively low before 2013 and steadily increased through 2024, as demonstrated in Figure 3. The United States outperformed all other countries, showing positive trends in the publications of studies on machine learning and health technologies in HIV testing from 2000 to 2024.

**Table 6.** Top 10 most relevant institutions by number of publications.

Institution	Articles, n (%)
University of California system	67
University of North Carolina	57
University of North Carolina Chapel Hill	52
University of Washington	48
University of Washington Seattle	48
Emory University	40
Yale University	35
University of Michigan	34
University of Michigan system	34
Johns Hopkins University	33

**Table 7.** Top 10 countries by scientific production.

Rank	Country	Articles, n (%)
1	United States of America	157 (59)
2	China	27 (10.2)
3	South Africa	16 (6)
4	United Kingdom	10 (3.8)
5	Australia	7 (2.6)
6	Canada	5 (1.9)
7	Switzerland	5 (1.9)
8	Kenya	4 (1.5)
9	India	3 (1.1)
10	The Netherlands	3 (1.1)

### Science Mapping

#### International Collaboration Networks

**Figure 4.**
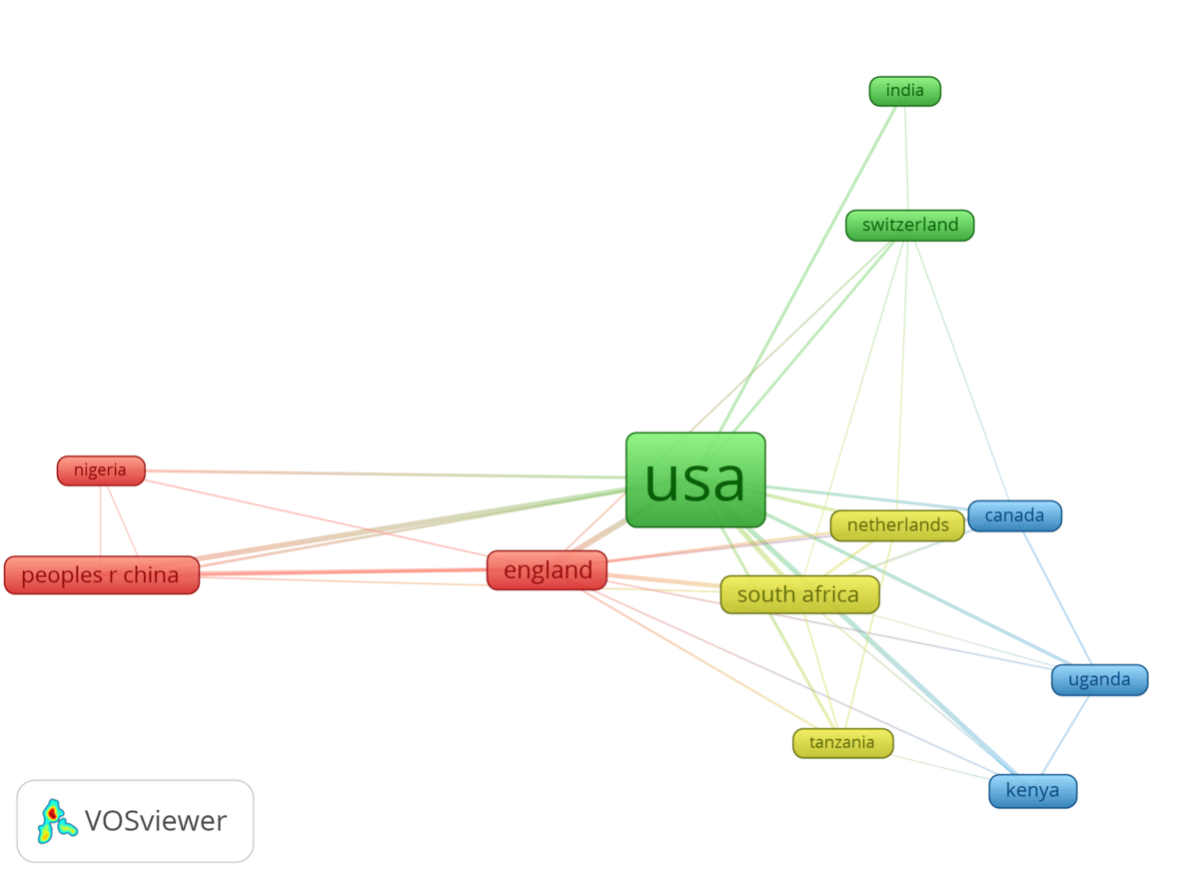
Country collaboration network. Peoples R China: People’s Republic of China; USA: United States of America.

Scientific collaborations between countries, institutions, and authors are shown at different levels. Figure 4 presents the most productive countries and their collaboration patterns when publishing studies focusing on machine learning in HIV testing. The country collaboration network shown in Figure 4 consisted of 13 productive countries, each with at least 5 documents. The network was divided into 6 clusters, wherein Canada, Rwanda, Switzerland, and Uganda are in cluster 1 (red). Cluster 2 (green) combines European and African countries such as Germany, the Netherlands, South Africa, and Tanzania. Australia, England, Nigeria, and China are in cluster 3 (blue), and Kenya, Peru, Thailand, and Zimbabwe are in cluster 4 (yellow). Cluster 5 (violet) comprises only European countries, including North Ireland, Norway, and Sweden. Cluster 6 (light blue) has 3 countries from different continents: Brazil, Malaysia, and the United States. The United States emerged as the most productive country, with a link strength of 118, and partnered with 100% of the countries, followed by England, which partnered with 77% of the 13 countries in the network. Australia and India have the lowest amount of country collaboration in studies applying machine learning and emerging health technologies in HIV testing (Figure 4).

**Figure 5.**
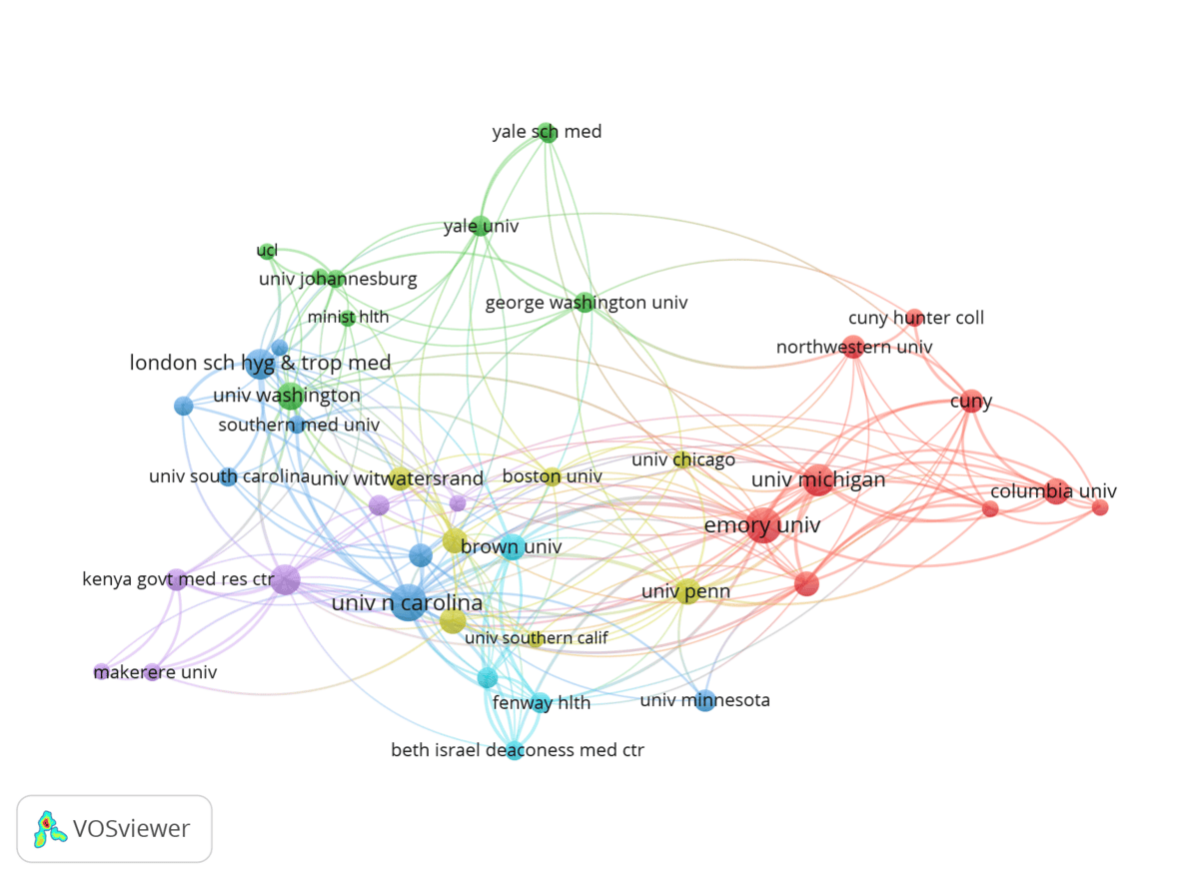
Institutional collaboration network.

Regarding the institutional collaboration network, each institution was set to include at least 5 documents, resulting in 43 productive universities (Figure 5). The most productive universities are in the United States. In terms of link strengths, the University of North Carolina ranked the highest (n=77), with 922 citations, followed by Emory University (n=62), the University of Michigan (n=45), San Diego State University (n=41), and the University of Pennsylvania (n=38). The University of Witwatersrand in South Africa had the highest link strength (n=7) among the African universities, with 158 citations. Among the top 16 universities, the University of North Carolina, Emory University, the University of Pennsylvania, and the University of Michigan have each collaborated with >60% of the institutions within the network. The only African university with good performance is the University of Witwatersrand, exhibiting 19% coauthorship within the network of 43 best universities writing papers on machine learning and emerging health technologies in HIV testing.

#### Co-Citation Analysis

**Figure 6.**
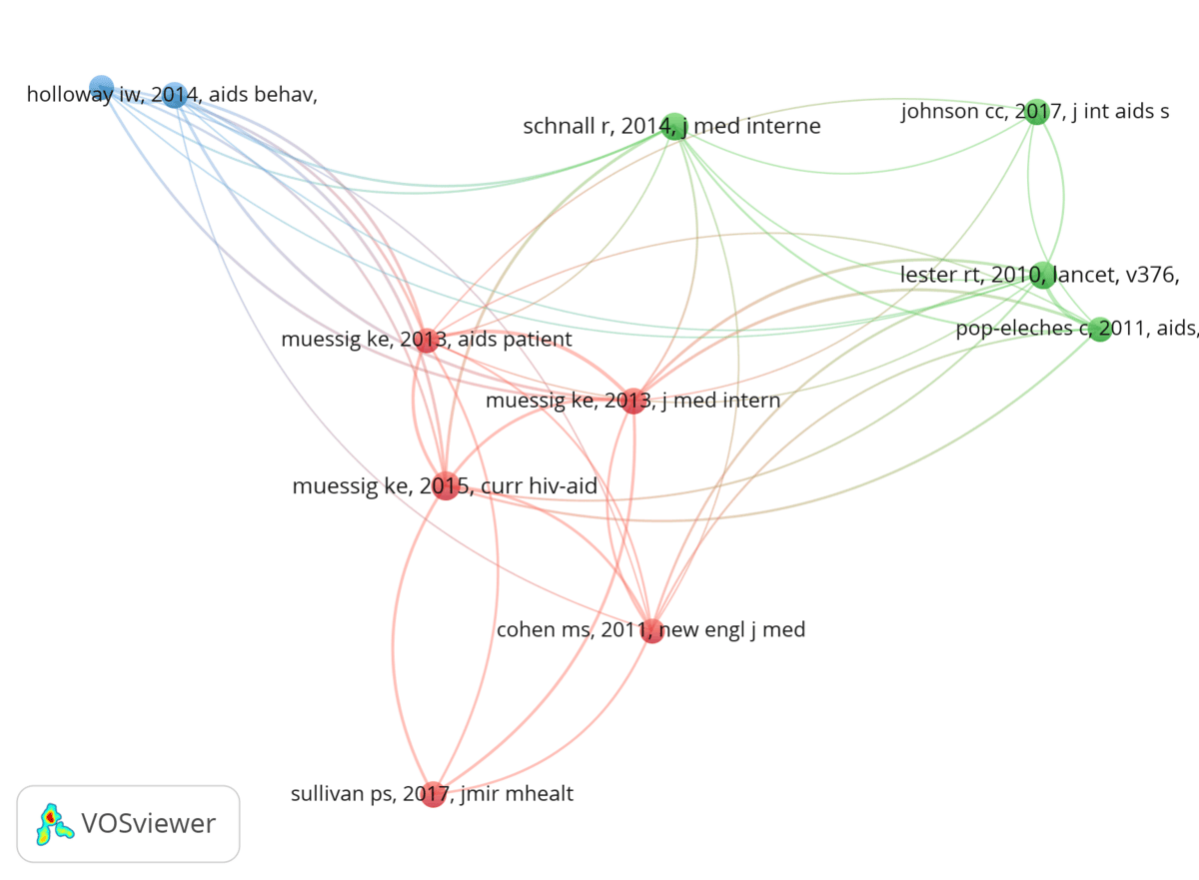
Network of co-cited references.

A network of co-citation analysis in Figure 6 shows the relationship among cited publications within the research area. The network was set to include only articles with a minimum of 15 citations, in which 11 documents met the threshold. Each article within the network is represented by a node, and the links between the nodes indicate co-cited studies, wherein the studies’ strengths and their co-citations are proportional to the nodes’ sizes and links’ thicknesses, respectively. The most cited document within the network was published by Muessig et al in 2015 [28] (25 citations), titled “A Systematic Review of Recent Smartphone, Internet, and Web 2.0 Interventions to Address the HIV Continuum of Care.” The study synthesized evidence from observational and experimental studies focused on understanding the dynamics of technology toward the HIV care cascade, including HIV testing. Three thematic clusters were produced within the network map of co-cited references (Figure 6). Cluster 1 (red) relates to studies on applying machine learning (technology) in HIV prevention in the context of testing. Cluster 2 (green) is associated with studies using machine learning (technology) in HIVST, and the last (blue) cluster within the network focused on the application of mHealth to improve HIV testing among men who have sex with men (MSM). The most globally cited documents are summarized in Table 5 with their digital object identifiers (DOI).

#### Occurrence of Authors’ Keywords

The analysis presents the most frequent keywords used by authors applying machine learning and emerging health technologies in HIV testing. The word cloud in Multimedia Appendix 2 shows the authors’ 50 most frequent keywords in this research domain. The word’s size in the cloud is proportional to the frequency at which it appears in the bibliographic sample. From the 50 frequently used keywords, HIV ranked the highest, with an occurrence of 93, followed by mHealth (n=65), MSM (n=46), HIV testing (n=41), HIV prevention (n=34), mobile phone (n=22), HIVST (n=21), and machine learning (n=20). These words established the thematic focus of the content analysis.

**Figure 7.**
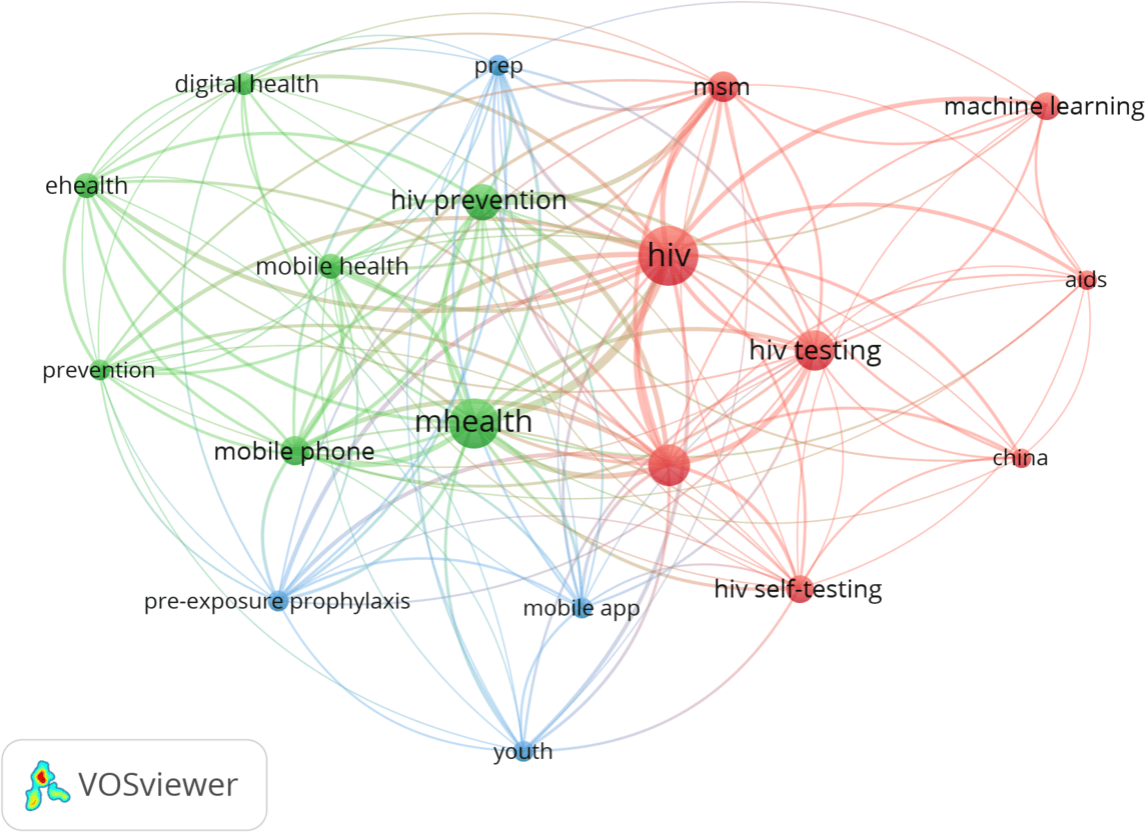
Co-occurrence network.

Figure 7 shows a network of co-occurrence of the authors’ most frequent keywords, providing an understanding of their interrelatedness. Using a threshold of 10 for the number of keyword occurrences, a network of the 19 most relevant keywords highlights that HIV has the highest link strength (n=177), with 100% co-occurrence of the 19 keywords. The following keywords also exhibit high link strengths in co-occurrences: mHealth, HIV prevention, MSM, HIV testing, and machine learning. A total of 3 clusters consisting of words within closely related topics were derived from the 19 author keywords within the co-word analysis (Figure 7). Cluster 1 (red) is related to the use of machine learning in HIV testing among MSM, with HIV, AIDS, HIV testing, HIVST, and MSM being the top keywords. Cluster 2 (green) is associated with mHealth and HIV prevention–related topics, and the top keywords are mHealth, digital health, mobile health, and HIV prevention. Likewise, the third cluster (blue), which encompasses keywords such as mobile app, youth, and pre-exposure prophylaxis, also relates to digital health and HIV prevention interventions among youths.

### Content Analysis

This bibliometric analysis explores studies that apply different machine learning techniques or technologies, including eHealth and mHealth, to improve HIV testing and provide insights into the research landscape. We reviewed the contents of 12 articles, with the most prominent authors’ keywords presented in Figure 7. The content analysis qualitatively summarized the key findings of the reviewed studies, focusing on the main concepts and topics constructed by the word cloudand co-occurrences (Figure 7) of authors’ keywords.

### The Application of Technology in HIV Prevention Among Priority Populations

Priority populations consist of young people and the different categories of KPs who are highly susceptible to HIV [29]. Based on the themes generated in the bibliometric analysis, we focus mainly on young people and MSM as key drivers of HIV due to societal stigma and discrimination, lack of HIV knowledge, and engaging in risky behaviors [29]. On top of that, their HIV testing uptake remains low, increasing the spread of the virus and the number of people living with HIV with unknown status [29]. Priority populations’ vulnerability to HIV makes them eligible for special interventions, and a variety of emerging technologies are being used to provide efficient and effective HIV prevention methods. We explored different studies addressing the gap within this thematic area. A machine learning technique predicted the internet health information–seeking behaviors of youths at risk of acquiring HIV by using their self-reported internet use data [30]. The study found that about 50% of the youths sought general health care information and sexual and reproductive health information, while 25% sought social services information [30]. In the same study, cisgender and transgender youths were less likely to seek health care information [30]. In another study conducted to predict HIV status among MSM in Zimbabwe, the authors applied different machine learning algorithms [31]. The findings showed that they could be used to enhance the early detection of HIV in order to establish targeted HIV testing for such hard-to-reach populations [31]. The two studies highlight the impact of societal stigma and that a lack of health care information impedes the success of HIV prevention and testing among young people and KPs and suggest the adoption of personalized HIV counseling and testing services [30,31]. Several machine learning and digital technologies, including those encompassed by eHealth and mHealth, have provided efficient HIV testing services by overcoming barriers related to privacy concerns among priority populations [32-37]. From identifying the most eligible candidates for HIV testing to enabling HIVST devices, these studies demonstrate how technology advancements have improved HIV prevention in the context of testing over the past 2 decades [32-37].

### Enhancing HIV Self-Testing Using Machine Learning and mHealth Solutions

Self-testing enables people to test for HIV and get their results at home or privately, offering an opportunity to overcome privacy concerns associated with HIV testing, especially among KPs [38]. This is a promising approach to scale up HIV testing but also raises ethical concerns regarding HIV counseling and testing. Advancements in machine learning and mHealth technologies have been used to address this challenge. A feasibility and acceptability assessment of HIVST using conversational agents in South Africa showed substantial progress in this approach [39]. The study developed chatbots for smartphones that were able to give written informed consent to the study participants, allowing them to self-counsel and test for HIV [39]. Fascinatingly, 79% of the users stated that their experiences with the chatbot were preferable to a human counselor [39]. A pragmatic trial involving refugee adolescents and youth in Uganda examined the feasibility of HIVST with and without mHealth [40]. The study participants, who were cisgender and transgender men and women, exhibited increased self-reported HIV testing uptake with the mHealth HIVST approach [40]. Another study [41] also emphasized the critical role mobile phones and digital technology can play in improving HIVST and linkage to care. Despite the opportunity to combat HIV testing barriers among KPs, a review of technology, policy, and evidence regarding HIVST in SSA reported that there is limited delivery within the region [42]. Most of these studies were piloted in Southern and Eastern Africa, necessitating future feasibility studies of HIVST in other African subregions.

## Discussion

### Principal Findings

This bibliometric analysis provides comprehensive insights into the evolution of machine learning and related technologies in HIV testing interventions from 2000 to 2024. By examining the trends and patterns of research outputs in this field over the past 2 decades, the study shows that publications were relatively low before 2010. The number of publications applying machine learning and emerging health technologies in HIV testing increased rapidly starting in 2013 and was highest in 2021, indicating research progress in this field during this period. The advancement of technology, among other factors, has contributed to the recent rapid growth of publications in this field. Recent breakthroughs in AI have led to increased acceptance in health care [43]. In the past decade, robotics, speech recognition, and machine learning have created new potential for disease prediction, diagnosis, and treatment [44,45]. Machine learning research in health care is expected to grow exponentially, leading to more publications in the future [46]. Our findings are consistent with a bibliometric analysis demonstrating the current growth of AI in health care research [46], and the authors project that the publication rate will double in the next 5 years. Moreover, the surge of publications in different public health domains, including HIV, especially in 2021, was driven by the COVID-19 pandemic, and our topic is no exception [47].

This study showed that the geographical distribution of the scientific production (articles) and collaboration were unequal. The United States ranked the highest, followed by China, South Africa, the United Kingdom, and Australia. These countries are home to top global universities and are well-known for their remarkable advancements in technology-driven medical research. Our analysis further emphasizes that academic collaborations on machine learning and health technologies in HIV testing are more common among universities in high-income countries. The University of North Carolina, Emory University, the University of Michigan, San Diego State University, and the University of Pennsylvania, all within the United States, exhibited the highest collaboration. The only African university that performed well was the University of Witwatersrand in South Africa. Unfortunately, low-income countries, which are disproportionately affected by the HIV epidemic, are missing out on the opportunity to leverage machine learning to enhance HIV testing. A similar study by Amusa et al [23] titled “Big Data and Infectious Disease in Epidemiology: Bibliometric Analysis and Research Agenda” revealed that institutions in advanced countries such as the United States and China contribute the most publications to this field. Previous studies also demonstrated increased academic collaboration among institutions in high-income countries [21,23,48], highlighting the need for greater equity in research partnerships.

The lack of collaboration within and across different settings may jeopardize knowledge translation into evidence-based programs and policies. This increases the need for support from the most productive countries (United States, European countries) to enhance the application of evidence on machine learning and emerging technologies in HIV testing in different contexts (eg, in SSA). Hence, productive universities and/or initiatives within SSA countries should foster collaborative research across different regions and continents in the future.

Subjects related to machine learning, technology, and HIV testing are usually published in public health–related journals, as shown in the analysis. The *Journal of AIDS and Behavior* recorded the highest h-index (10), with 494 citations. *The Journal of Medical Internet Research*, *JMIR mHealth and uHealth*, *JMIR Research Protocols*, *mHealth*, *AIDS Care-Psychological and Socio-Medical Aspects of AI*, *BMC Public Health*, and *PLOS One* were also very influential in publishing studies on machine learning and health technologies in HIV testing. Similar findings were reported in bibliometric analyses focused on digital technologies and HIV research [21,22,48,49].

In analyzing the authors’ keywords, we identified themes and topics based on the frequency and co-occurrence of specific words. Keywords that appeared together within the same cluster indicated related topics. Frequent keywords such as HIV, HIV prevention, MSM, HIVST, HIV testing, mHealth, mobile app, youth, digital health, eHealth, and machine learning appeared in almost all the clusters in the co-word analysis, resulting in 2 thematic topics. These themes reflect the current focus of this field and suggest future research on machine learning and technology in connection with HIV testing. The content analysis, which focused mainly on the application of machine learning and digital technologies in HIV prevention, including HIVST among KPs, gave comprehensive insights into the extent to which such technologies have enhanced HIV testing and identified limitations as well as suggestions for future studies. The analysis revealed that machine learning and digital technologies can significantly enhance HIV testing among priority groups, including young people and KPs like transgender people, cisgender people, and MSM. Key populations are becoming the center of focus when designing and implementing HIV prevention interventions [29-31]. Such groups are hard to reach due to societal stigma and legal issues, especially in SSA countries [29-31].

A recent systematic review highlighted that machine learning has significant potential to address privacy and accessibility gaps in HIV testing, particularly among KPs [50]. Although machine learning has proven to be highly accurate and effective in HIV testing, the study indicated that most interventions have focused on the Americas, as well as East and Southern Africa [50]. Consistently, our findings showed that machine learning has been used to predict health-seeking behaviors of high-risk populations [30], predict their HIV risk [31], and develop various options aimed at overcoming barriers to KPs accessing and using HIV testing. Among the developments, HIVST was identified as a viable option for conducting self-counseling and testing for HIV among KPs [38-42]. However, feasibility studies for machine learning and emerging technologies in HIV testing approaches have been piloted in only a few Southern and Eastern African countries. Furthermore, lack of expertise and inadequate infrastructure limit the application of advanced technologies and AI in HIV testing across many African countries [50]. This gap could leave the continent behind in meeting the UNAIDS 95-95-95 target despite bearing the brunt of the global HIV burden. Thus, these innovative techniques are essential for strengthening and should be implemented across SSA beyond piloting purposes.

### Limitations

This bibliometric analysis encountered several limitations. First, the WoS database was used to gather articles, which might have excluded relevant studies from other databases. The WoS is a leading database for studies on machine learning and technology in health care and has been recognized as a legitimate platform for bibliometric analysis [18-23]. Second, we only selected studies published in English, potentially missing relevant studies published in other languages. Third, our sample size was relatively small and may not fully represent research outputs in machine learning and technology applications in HIV testing. However, our findings are relevant as they are consistent with publication trends in similar bibliometric analyses. Moreover, the Biblioshiny software could not determine authors’ valid affiliations; a single author could have multiple affiliations in some instances. We chose the “Application Name Disambiguation” option to ensure the same affiliations were counted as one.

### Future Research and Conclusions

This study showed that universities in high-income countries like the United States, the United Kingdom, and South Africa are very productive in applying machine learning technology to address health care issues, including HIV testing. Journals publishing articles on public health and infectious diseases were very influential in this analysis. The most frequent keywords in the sample were related to the application of digital technologies in HIV prevention, particularly HIV testing among KPs. Furthermore, the study found that incorporating machine learning in HIVST seems promising for the enhancement of HIV testing among KPs, especially MSM. These insights demonstrate improved academic engagement and productivity over the 2 decades, identifying opportunities and research gaps for future scholars to embark on.

This bibliometric analysis highlights a research gap between high-income and underresourced nations regarding applying machine learning and related technologies in HIV testing interventions. More than half of the studies analyzed were from the United States, and a handful of them came from other advanced countries. Many low-income countries, especially in SSA, are lagging in using advanced technologies to tackle health care challenges. There is a critical need to develop an enabling infrastructure that can facilitate the use of machine learning in HIV testing interventions. Furthermore, academic institutions should incorporate machine learning courses to equip future researchers with the essential skills needed in this area. Bridging the intercontinental gap is crucial for overcoming the barriers to HIV prevention interventions, ultimately contributing to the success of UNAIDS’ goals to eradicate HIV/AIDS as a global health issue.

This bibliometric analysis clearly demonstrates a significant shift from conventional HIV testing methods to innovative approaches like HIVST. The WHO strongly endorses HIVST as an effective way to deliver personalized and context-specific HIV services that tackle the structural issues surrounding stigma and discrimination. Notably, both youth and KPs have expressed a clear preference for HIVST and mobile technology, which are crucial for improving linkage to care. Although HIVST is user-friendly and allows individuals to test conveniently at home, it presents a challenge in accurately monitoring national HIV testing rates. To address this issue, it is imperative that HIV policies implement robust tracking systems to ensure that individuals using home testing are included in national HIV testing data. This proactive approach is essential for effectively assessing and improving HIV care and services.

Machine learning models continually evolve to address stigma and discrimination, as well as privacy concerns related to HIV testing, proving to be efficient and effective despite implementation challenges. Future studies should assess the feasibility of various machine learning techniques to enhance HIV testing in low-income countries. Such insights will further identify solutions to the current gaps in research in low-income countries, particularly those in SSA. The study also highlights the need to improve collaboration among African universities to enhance research outputs using advanced methodologies. In today’s world, utilizing AI, machine learning, and advanced technologies is essential for addressing critical challenges across all sectors, especially health care. Based on the gaps and opportunities highlighted in this study, the findings would inform program implementers to design more effective strategies for improving the use of machine learning and technology in HIV testing. By increasing the use of machine learning and emerging technologies in HIV testing interventions, we can significantly boost the number of individuals who get tested for HIV. This will contribute to the success of the UNAIDS 2030 goal and result in improved health outcomes for our communities.

## Supplementary material

10.2196/64829 Multimedia Appendix 1 Most relevant countries.

10.2196/64829 Multimedia Appendix 2 Word cloud of authors’ keywords.

## Abbreviations

AI: artificial intelligence
DOI: digital object identifier
HIVST: HIV self-testing
KP: key population
MSM: men who have sex with men
PACER: Pan African Centre for Epidemics Research
RF: random forest
SAMRC: South African Medical Research Council
SSA: sub-Saharan Africa
UJ: University of Johannesburg
UNAIDS: The Joint United Nations Programme on HIV/AIDS
WHO: World Health Organization
WoS: Web of Science
XGBoost: Extreme Gradient Boosting
