# Supplementary figures and images for: Application of Machine Learning and Emerging Health Technologies in the Uptake of HIV Testing: Bibliometric Analysis of Studies Published From 2000 to 2024
Source: Interact J Med Res. 2025 May 22;14:e64829. doi: 10.2196/64829 (PMC12121542; doi:10.2196/64829)

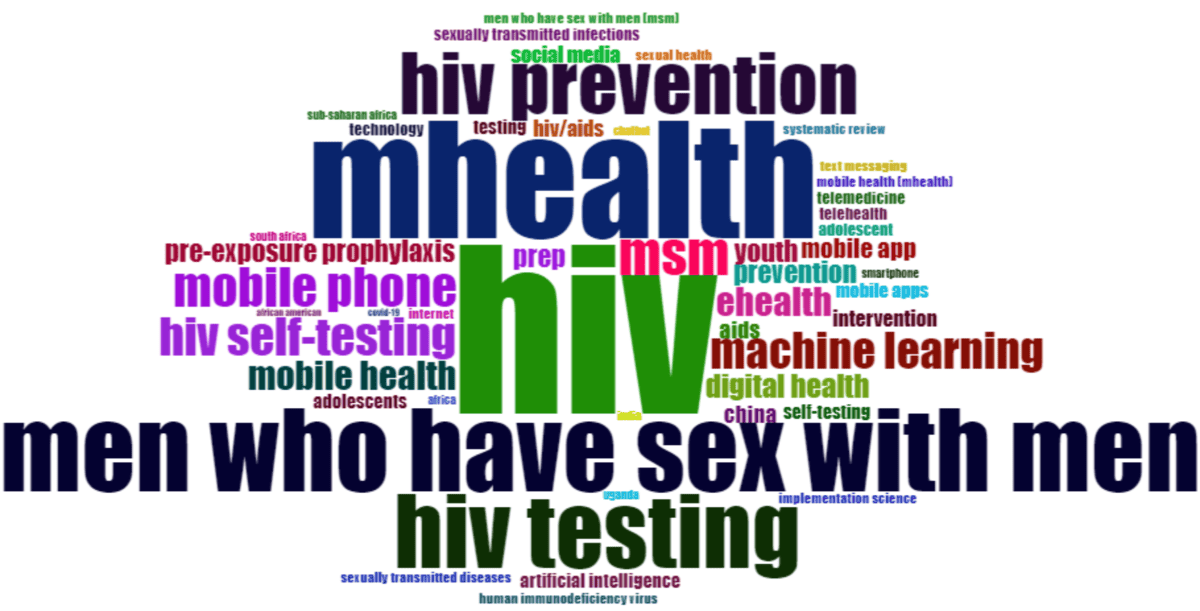

Supplement: Multimedia Appendix 2 [file ijmr-v14-e64829-s002.png]
